# Supplementary material for: Hot spot prediction in protein-protein interactions by an ensemble system
Source: BMC Syst Biol. 2018 Dec 31;12(Suppl 9):132. doi: 10.1186/s12918-018-0665-8 (PMC6311905; doi:10.1186/s12918-018-0665-8)
Supplement: Supplementary file 2 — All relevant MATLAB code. A simple Matlab implement of our predictor. (ZIP 19,742 kb) [file 12918_2018_665_MOESM2_ESM.zip › Additional file 2/Read ASA/ASA website/dbMPIKT/1S78A.html]

 NetSurfP - Protein Surface Accessibility and Secondary Structure Predictions

  

|  |  |  |
| --- | --- | --- |
|  |  | NetSurfP - Protein Surface Accessibility and Secondary Structure Predictions  Technical University of Denmark |

  


---

```
# For publication of results, please cite:
# A generic method for assignment of reliability scores applied to solvent accessibility predictions.
# Bent Petersen, Thomas Nordahl Petersen, Pernille Andersen, Morten Nielsen and Claus Lundegaard
# BMC Structural Biology 2009, 9:51 doi:10.1186/1472-6807-9-51
#
# Column 1: Class assignment - B for buried or E for Exposed - Threshold: 25% exposure, but not based on RSA
# Column 2: Amino acid
# Column 3: Sequence name
# Column 4: Amino acid number
# Column 5: Relative Surface Accessibility - RSA
# Column 6: Absolute Surface Accessibility
# Column 7: Z-fit score for RSA prediction
# Column 8: Probability for Alpha-Helix
# Column 9: Probability for Beta-strand
# Column 10: Probability for Coil
E T  1S78A                  1    0.774 107.298  -0.177   0.003   0.003   0.994
E Q  1S78A                  2    0.517  92.300   0.712   0.004   0.420   0.576
E V  1S78A                  3    0.379  58.191   0.288   0.004   0.420   0.576
B C  1S78A                  4    0.058   8.115  -1.259   0.005   0.262   0.733
E T  1S78A                  5    0.548  75.938   0.331   0.021   0.279   0.699
E G  1S78A                  6    0.419  32.952  -1.699   0.019   0.141   0.840
E T  1S78A                  7    0.335  46.520   0.285   0.019   0.141   0.840
E D  1S78A                  8    0.467  67.324  -1.246   0.056   0.142   0.802
B M  1S78A                  9    0.201  40.180  -1.326   0.052   0.084   0.864
E K  1S78A                 10    0.402  82.774  -0.892   0.113   0.087   0.800
B L  1S78A                 11    0.207  37.957  -1.800   0.052   0.084   0.864
E R  1S78A                 12    0.407  93.249  -0.686   0.052   0.084   0.864
E L  1S78A                 13    0.285  52.110  -0.494   0.018   0.047   0.935
E P  1S78A                 14    0.467  66.267  -1.525   0.018   0.047   0.935
E A  1S78A                 15    0.539  59.354  -2.849   0.018   0.019   0.964
E S  1S78A                 16    0.395  46.271  -1.980   0.018   0.019   0.964
E P  1S78A                 17    0.341  48.445  -1.378   0.321   0.003   0.675
E E  1S78A                 18    0.599 104.593  -0.562   0.600   0.003   0.397
E T  1S78A                 19    0.421  58.379  -0.160   0.717   0.014   0.269
B H  1S78A                 20    0.173  31.505   0.281   0.858   0.002   0.139
B L  1S78A                 21    0.134  24.572  -0.023   0.923   0.002   0.076
E D  1S78A                 22    0.560  80.754   0.957   0.970   0.001   0.030
E M  1S78A                 23    0.305  61.051   0.593   0.970   0.001   0.030
B L  1S78A                 24    0.044   8.093   0.207   0.970   0.001   0.030
E R  1S78A                 25    0.355  81.387   1.118   0.923   0.002   0.076
E H  1S78A                 26    0.494  89.950   0.927   0.923   0.002   0.076
B L  1S78A                 27    0.165  30.230   0.323   0.858   0.002   0.139
B Y  1S78A                 28    0.079  16.861  -0.337   0.502   0.002   0.495
E Q  1S78A                 29    0.571 101.945   0.885   0.109   0.005   0.886
E G  1S78A                 30    0.528  41.561  -0.030   0.018   0.019   0.964
B C  1S78A                 31    0.037   5.223   0.777   0.004   0.197   0.799
E Q  1S78A                 32    0.293  52.330   0.426   0.002   0.816   0.182
B V  1S78A                 33    0.224  34.490   1.295   0.001   0.959   0.040
B V  1S78A                 34    0.075  11.466   0.843   0.001   0.959   0.040
B Q  1S78A                 35    0.273  48.669   0.058   0.001   0.900   0.099
B G  1S78A                 36    0.110   8.633  -1.454   0.002   0.816   0.182
B N  1S78A                 37    0.254  37.200  -0.368   0.001   0.900   0.099
B L  1S78A                 38    0.074  13.586   0.587   0.001   0.900   0.099
B E  1S78A                 39    0.193  33.700   0.421   0.001   0.959   0.040
B L  1S78A                 40    0.033   5.987   0.376   0.001   0.959   0.040
B T  1S78A                 41    0.167  23.191   0.876   0.011   0.918   0.071
B Y  1S78A                 42    0.121  25.922   0.151   0.002   0.816   0.182
B L  1S78A                 43    0.103  18.804   0.397   0.004   0.514   0.481
E P  1S78A                 44    0.388  55.086  -0.866   0.019   0.141   0.840
E T  1S78A                 45    0.592  82.124  -0.552   0.018   0.088   0.893
E N  1S78A                 46    0.461  67.549  -0.617   0.018   0.047   0.935
E A  1S78A                 47    0.553  60.985   0.124   0.018   0.019   0.964
E S  1S78A                 48    0.631  73.918  -0.039   0.018   0.019   0.964
B L  1S78A                 49    0.155  28.381   0.091   0.018   0.047   0.935
E S  1S78A                 50    0.600  70.320  -0.851   0.053   0.043   0.903
E F  1S78A                 51    0.257  51.580   0.613   0.113   0.043   0.844
B L  1S78A                 52    0.091  16.662  -0.161   0.115   0.016   0.868
E Q  1S78A                 53    0.565 100.873   0.777   0.115   0.016   0.868
E D  1S78A                 54    0.486  70.076   1.242   0.184   0.043   0.773
B I  1S78A                 55    0.062  11.470   0.689   0.199   0.152   0.649
E Q  1S78A                 56    0.356  63.510   0.899   0.159   0.516   0.325
E E  1S78A                 57    0.400  69.845   0.715   0.087   0.683   0.230
B V  1S78A                 58    0.072  11.066   0.649   0.085   0.775   0.140
B Q  1S78A                 59    0.218  38.899   0.436   0.074   0.484   0.442
B G  1S78A                 60    0.191  15.008  -1.608   0.021   0.451   0.528
B Y  1S78A                 61    0.093  19.831   0.919   0.018   0.846   0.136
B V  1S78A                 62    0.027   4.150   0.828   0.011   0.918   0.071
B L  1S78A                 63    0.061  11.261   1.008   0.006   0.962   0.032
B I  1S78A                 64    0.022   4.070   0.850   0.011   0.918   0.071
B A  1S78A                 65    0.042   4.639  -0.153   0.011   0.918   0.071
B H  1S78A                 66    0.182  33.069   0.027   0.021   0.756   0.223
B N  1S78A                 67    0.077  11.317  -0.856   0.022   0.359   0.619
E Q  1S78A                 68    0.449  80.138  -0.233   0.064   0.216   0.721
B V  1S78A                 69    0.246  37.749  -1.535   0.056   0.142   0.802
E R  1S78A                 70    0.439 100.623  -0.643   0.056   0.142   0.802
E Q  1S78A                 71    0.486  86.889  -0.390   0.019   0.141   0.840
E V  1S78A                 72    0.371  57.007   0.052   0.056   0.142   0.802
E P  1S78A                 73    0.350  49.708  -0.065   0.052   0.084   0.864
B L  1S78A                 74    0.057  10.400  -0.101   0.053   0.043   0.903
E Q  1S78A                 75    0.585 104.535   0.335   0.018   0.047   0.935
E R  1S78A                 76    0.427  97.806  -0.410   0.018   0.047   0.935
B L  1S78A                 77    0.084  15.380   0.555   0.020   0.205   0.775
E R  1S78A                 78    0.357  81.822   0.104   0.023   0.655   0.322
B I  1S78A                 79    0.226  41.866  -0.102   0.021   0.756   0.223
B V  1S78A                 80    0.073  11.235   0.413   0.021   0.756   0.223
E R  1S78A                 81    0.342  78.364  -0.048   0.021   0.451   0.528
B G  1S78A                 82    0.282  22.170  -1.919   0.019   0.141   0.840
E T  1S78A                 83    0.444  61.638   0.232   0.022   0.359   0.619
E Q  1S78A                 84    0.418  74.708   0.160   0.023   0.655   0.322
B L  1S78A                 85    0.228  41.765   0.325   0.023   0.655   0.322
B F  1S78A                 86    0.189  37.952   0.366   0.079   0.592   0.329
E E  1S78A                 87    0.449  78.370  -0.441   0.069   0.386   0.545
E D  1S78A                 88    0.413  59.557  -1.178   0.069   0.386   0.545
E N  1S78A                 89    0.322  47.170  -0.012   0.021   0.451   0.528
B Y  1S78A                 90    0.070  14.916  -0.071   0.021   0.756   0.223
B A  1S78A                 91    0.035   3.890  -0.813   0.011   0.918   0.071
B L  1S78A                 92    0.035   6.482  -0.226   0.011   0.918   0.071
B A  1S78A                 93    0.034   3.714   0.187   0.006   0.962   0.032
B V  1S78A                 94    0.049   7.485  -0.504   0.011   0.918   0.071
B L  1S78A                 95    0.066  12.103  -0.802   0.018   0.846   0.136
B D  1S78A                 96    0.204  29.425  -1.064   0.023   0.655   0.322
B N  1S78A                 97    0.139  20.379  -0.795   0.022   0.359   0.619
E G  1S78A                 98    0.345  27.167  -0.796   0.019   0.141   0.840
E D  1S78A                 99    0.443  63.793  -0.985   0.018   0.088   0.893
E P  1S78A                100    0.399  56.604  -1.252   0.052   0.084   0.864
E L  1S78A                101    0.411  75.181  -2.442   0.056   0.142   0.802
E N  1S78A                102    0.370  54.124  -0.798   0.118   0.150   0.732
B N  1S78A                103    0.226  33.086  -2.532   0.118   0.150   0.732
E T  1S78A                104    0.325  45.105  -1.933   0.118   0.150   0.732
E T  1S78A                105    0.362  50.251  -1.649   0.125   0.227   0.648
B P  1S78A                106    0.247  35.078  -1.549   0.125   0.227   0.648
B V  1S78A                107    0.291  44.696  -1.546   0.125   0.227   0.648
B T  1S78A                108    0.248  34.411  -1.541   0.135   0.317   0.548
E G  1S78A                109    0.314  24.751  -2.073   0.135   0.317   0.548
E A  1S78A                110    0.408  44.918  -1.470   0.216   0.235   0.548
E S  1S78A                111    0.397  46.517  -1.790   0.118   0.150   0.732
E P  1S78A                112    0.442  62.677  -1.195   0.118   0.150   0.732
E G  1S78A                113    0.324  25.491  -0.943   0.056   0.142   0.802
E G  1S78A                114    0.286  22.532   0.058   0.066   0.296   0.638
B L  1S78A                115    0.075  13.787  -0.634   0.022   0.552   0.426
E R  1S78A                116    0.320  73.188   0.068   0.021   0.756   0.223
E E  1S78A                117    0.348  60.848  -0.382   0.021   0.756   0.223
B L  1S78A                118    0.091  16.625   0.459   0.023   0.655   0.322
E Q  1S78A                119    0.420  74.941  -0.479   0.021   0.451   0.528
B L  1S78A                120    0.074  13.586  -0.230   0.020   0.205   0.775
E R  1S78A                121    0.487 111.431   0.249   0.018   0.088   0.893
E S  1S78A                122    0.566  66.277   0.429   0.052   0.084   0.864
B L  1S78A                123    0.169  30.944  -0.404   0.191   0.086   0.723
E T  1S78A                124    0.446  61.832   0.073   0.199   0.152   0.649
E E  1S78A                125    0.386  67.364   1.256   0.231   0.330   0.439
B I  1S78A                126    0.054  10.045   0.480   0.231   0.330   0.439
E L  1S78A                127    0.299  54.820  -0.275   0.135   0.317   0.548
E K  1S78A                128    0.454  93.470   0.217   0.021   0.279   0.699
B G  1S78A                129    0.108   8.507  -1.586   0.004   0.138   0.858
B G  1S78A                130    0.134  10.522  -1.080   0.004   0.420   0.576
B V  1S78A                131    0.053   8.208  -1.092   0.001   0.900   0.099
B L  1S78A                132    0.153  27.941   0.770   0.001   0.959   0.040
B I  1S78A                133    0.028   5.143  -0.751   0.001   0.959   0.040
B Q  1S78A                134    0.175  31.219  -0.598   0.001   0.900   0.099
E R  1S78A                135    0.370  84.753  -0.459   0.004   0.514   0.481
B N  1S78A                136    0.156  22.897  -0.424   0.005   0.045   0.951
E P  1S78A                137    0.468  66.409  -1.262   0.018   0.088   0.893
E Q  1S78A                138    0.443  79.209  -1.008   0.022   0.359   0.619
B L  1S78A                139    0.161  29.534  -1.376   0.021   0.451   0.528
B C  1S78A                140    0.202  28.375  -1.350   0.022   0.552   0.426
E Y  1S78A                141    0.419  89.519  -1.648   0.021   0.451   0.528
E Q  1S78A                142    0.372  66.457  -1.113   0.021   0.279   0.699
E D  1S78A                143    0.347  49.945  -1.397   0.005   0.262   0.733
E T  1S78A                144    0.488  67.616  -0.674   0.020   0.205   0.775
B I  1S78A                145    0.344  63.603  -1.857   0.053   0.043   0.903
E L  1S78A                146    0.394  72.233  -0.660   0.115   0.016   0.868
B W  1S78A                147    0.136  32.828  -0.546   0.455   0.046   0.498
E K  1S78A                148    0.505 103.899   0.664   0.455   0.046   0.498
E D  1S78A                149    0.309  44.498   0.092   0.502   0.102   0.396
B I  1S78A                150    0.065  12.099   0.158   0.538   0.173   0.289
B F  1S78A                151    0.141  28.279  -0.547   0.453   0.248   0.299
E H  1S78A                152    0.487  88.658  -0.164   0.199   0.152   0.649
E K  1S78A                153    0.467  96.103  -0.428   0.053   0.043   0.903
E N  1S78A                154    0.486  71.224  -1.851   0.018   0.019   0.964
E N  1S78A                155    0.606  88.762  -2.316   0.018   0.019   0.964
E Q  1S78A                156    0.561 100.177  -0.790   0.018   0.047   0.935
B L  1S78A                157    0.243  44.493  -1.788   0.004   0.085   0.910
E A  1S78A                158    0.350  38.603  -1.243   0.005   0.336   0.660
E L  1S78A                159    0.408  74.650  -1.798   0.004   0.616   0.381
E T  1S78A                160    0.416  57.769  -1.462   0.003   0.718   0.279
E L  1S78A                161    0.280  51.250  -0.980   0.021   0.756   0.223
B I  1S78A                162    0.266  49.191  -0.535   0.023   0.655   0.322
E D  1S78A                163    0.436  62.856  -1.217   0.021   0.451   0.528
E T  1S78A                164    0.500  69.308  -1.038   0.020   0.205   0.775
E N  1S78A                165    0.632  92.539  -1.986   0.018   0.047   0.935
E R  1S78A                166    0.543 124.347  -1.194   0.018   0.047   0.935
E S  1S78A                167    0.568  66.570  -0.935   0.004   0.138   0.858
E R  1S78A                168    0.404  92.493  -0.037   0.005   0.336   0.660
E A  1S78A                169    0.458  50.472   0.624   0.004   0.420   0.576
B C  1S78A                170    0.103  14.503  -1.596   0.005   0.262   0.733
E H  1S78A                171    0.609 110.759   0.074   0.005   0.262   0.733
E P  1S78A                172    0.657  93.242   0.760   0.004   0.138   0.858
B C  1S78A                173    0.138  19.431  -0.325   0.018   0.019   0.964
E S  1S78A                174    0.506  59.268  -0.532   0.018   0.019   0.964
E P  1S78A                175    0.703  99.784  -0.603   0.018   0.019   0.964
E M  1S78A                176    0.617 123.482  -0.070   0.018   0.019   0.964
B C  1S78A                177    0.105  14.798  -1.412   0.018   0.019   0.964
E K  1S78A                178    0.634 130.352  -0.083   0.018   0.047   0.935
E G  1S78A                179    0.634  49.904  -0.376   0.004   0.138   0.858
E S  1S78A                180    0.387  45.368  -0.007   0.005   0.262   0.733
E R  1S78A                181    0.458 104.790  -0.213   0.004   0.514   0.481
B C  1S78A                182    0.083  11.723  -3.314   0.004   0.616   0.381
E W  1S78A                183    0.384  92.376  -1.242   0.004   0.514   0.481
E G  1S78A                184    0.443  34.872  -1.773   0.004   0.197   0.799
E E  1S78A                185    0.565  98.705  -1.474   0.005   0.045   0.951
E S  1S78A                186    0.597  70.004  -1.528   0.018   0.019   0.964
E S  1S78A                187    0.387  45.403  -0.503   0.018   0.019   0.964
E E  1S78A                188    0.576 100.645  -1.092   0.053   0.043   0.903
E D  1S78A                189    0.410  59.081   0.384   0.056   0.142   0.802
B C  1S78A                190    0.109  15.276  -3.020   0.066   0.296   0.638
E Q  1S78A                191    0.301  53.705  -0.681   0.021   0.451   0.528
E S  1S78A                192    0.402  47.161  -1.600   0.021   0.451   0.528
E L  1S78A                193    0.343  62.767  -0.612   0.022   0.359   0.619
E T  1S78A                194    0.378  52.442  -0.932   0.021   0.279   0.699
E R  1S78A                195    0.651 148.987  -0.704   0.004   0.138   0.858
E T  1S78A                196    0.382  52.970  -1.706   0.005   0.262   0.733
E V  1S78A                197    0.485  74.544  -0.248   0.022   0.552   0.426
B C  1S78A                198    0.078  10.895  -2.274   0.022   0.552   0.426
E A  1S78A                199    0.422  46.482  -0.513   0.022   0.359   0.619
E G  1S78A                200    0.545  42.876  -1.560   0.020   0.205   0.775
E G  1S78A                201    0.624  49.109  -1.469   0.018   0.088   0.893
B C  1S78A                202    0.276  38.750  -0.971   0.018   0.088   0.893
E A  1S78A                203    0.444  48.951  -1.900   0.019   0.141   0.840
E R  1S78A                204    0.505 115.576  -0.869   0.020   0.205   0.775
E C  1S78A                205    0.374  52.552  -1.995   0.021   0.279   0.699
E K  1S78A                206    0.446  91.804  -1.183   0.021   0.279   0.699
E G  1S78A                207    0.348  27.427  -2.106   0.020   0.205   0.775
E P  1S78A                208    0.532  75.548  -0.803   0.019   0.141   0.840
E L  1S78A                209    0.562 102.939  -1.154   0.019   0.141   0.840
E P  1S78A                210    0.502  71.205  -0.082   0.004   0.197   0.799
E T  1S78A                211    0.420  58.212  -0.479   0.004   0.197   0.799
E D  1S78A                212    0.476  68.520   0.104   0.005   0.262   0.733
E C  1S78A                213    0.357  50.081   0.087   0.004   0.197   0.799
B C  1S78A                214    0.098  13.815  -0.039   0.018   0.047   0.935
E H  1S78A                215    0.351  63.774   0.435   0.018   0.019   0.964
E E  1S78A                216    0.621 108.454  -0.629   0.053   0.005   0.942
E Q  1S78A                217    0.534  95.390   0.352   0.058   0.017   0.925
B C  1S78A                218    0.073  10.263  -1.428   0.115   0.016   0.868
E A  1S78A                219    0.456  50.251  -0.309   0.113   0.043   0.844
E A  1S78A                220    0.588  64.754  -0.565   0.113   0.087   0.800
E G  1S78A                221    0.418  32.881  -0.327   0.056   0.142   0.802
B C  1S78A                222    0.107  14.967  -1.815   0.020   0.205   0.775
E T  1S78A                223    0.583  80.848  -1.259   0.020   0.205   0.775
E G  1S78A                224    0.454  35.706  -0.707   0.005   0.045   0.951
E P  1S78A                225    0.618  87.666  -1.483   0.003   0.003   0.994
E K  1S78A                226    0.542 111.531  -0.125   0.003   0.003   0.994
E H  1S78A                227    0.535  97.280  -0.226   0.005   0.015   0.979
E S  1S78A                228    0.489  57.276  -0.360   0.018   0.019   0.964
E D  1S78A                229    0.372  53.548   1.028   0.020   0.205   0.775
B C  1S78A                230    0.043   5.967  -1.228   0.021   0.451   0.528
E L  1S78A                231    0.271  49.638   0.517   0.021   0.756   0.223
E A  1S78A                232    0.402  44.256   0.387   0.023   0.655   0.322
B C  1S78A                233    0.063   8.803  -0.238   0.022   0.359   0.619
E L  1S78A                234    0.485  88.730   0.235   0.019   0.141   0.840
E H  1S78A                235    0.485  88.222  -0.781   0.019   0.141   0.840
B F  1S78A                236    0.251  50.376  -0.927   0.020   0.205   0.775
E N  1S78A                237    0.437  63.962  -0.831   0.019   0.141   0.840
E H  1S78A                238    0.518  94.261  -1.451   0.018   0.088   0.893
E S  1S78A                239    0.704  82.556  -0.622   0.005   0.015   0.979
E G  1S78A                240    0.477  37.508  -1.274   0.005   0.045   0.951
E I  1S78A                241    0.424  78.421   0.794   0.004   0.420   0.576
B C  1S78A                242    0.056   7.876  -0.769   0.003   0.718   0.279
E E  1S78A                243    0.343  59.835   0.479   0.021   0.756   0.223
E L  1S78A                244    0.452  82.743  -0.805   0.023   0.655   0.322
E H  1S78A                245    0.489  88.985   0.413   0.022   0.359   0.619
B C  1S78A                246    0.064   8.958  -0.784   0.004   0.085   0.910
E P  1S78A                247    0.505  71.631  -0.802   0.005   0.015   0.979
E A  1S78A                248    0.574  63.222  -0.871   0.018   0.047   0.935
E L  1S78A                249    0.461  84.372  -1.908   0.019   0.141   0.840
E V  1S78A                250    0.384  59.005  -0.785   0.021   0.451   0.528
B T  1S78A                251    0.171  23.704  -1.482   0.022   0.552   0.426
E Y  1S78A                252    0.411  87.831  -0.710   0.021   0.451   0.528
E N  1S78A                253    0.400  58.545  -0.717   0.021   0.279   0.699
E T  1S78A                254    0.629  87.215  -1.246   0.018   0.047   0.935
E D  1S78A                255    0.724 104.314  -1.239   0.004   0.085   0.910
E T  1S78A                256    0.453  62.887   0.321   0.005   0.336   0.660
E F  1S78A                257    0.281  56.437   0.625   0.003   0.718   0.279
E E  1S78A                258    0.310  54.122   0.575   0.018   0.846   0.136
B S  1S78A                259    0.212  24.858  -0.438   0.021   0.756   0.223
B M  1S78A                260    0.264  52.826   0.129   0.023   0.655   0.322
E P  1S78A                261    0.534  75.817  -0.297   0.021   0.279   0.699
E N  1S78A                262    0.282  41.314  -0.315   0.018   0.019   0.964
E P  1S78A                263    0.768 109.022  -0.667   0.016   0.005   0.979
E E  1S78A                264    0.664 116.053  -0.244   0.018   0.047   0.935
E G  1S78A                265    0.253  19.927  -0.426   0.019   0.141   0.840
E R  1S78A                266    0.456 104.401  -0.009   0.005   0.262   0.733
E Y  1S78A                267    0.323  69.111  -0.301   0.022   0.359   0.619
E T  1S78A                268    0.353  49.003  -1.109   0.020   0.205   0.775
E F  1S78A                269    0.509 102.156  -1.353   0.004   0.085   0.910
E G  1S78A                270    0.638  50.218  -0.616   0.005   0.015   0.979
E A  1S78A                271    0.637  70.252  -1.416   0.016   0.005   0.979
E S  1S78A                272    0.620  72.629   0.160   0.018   0.047   0.935
E C  1S78A                273    0.221  31.014  -0.463   0.021   0.279   0.699
B V  1S78A                274    0.191  29.311   0.127   0.022   0.359   0.619
E T  1S78A                275    0.495  68.615  -0.503   0.021   0.279   0.699
E A  1S78A                276    0.487  53.645  -0.038   0.020   0.205   0.775
B C  1S78A                277    0.091  12.819  -0.046   0.018   0.047   0.935
E P  1S78A                278    0.519  73.646  -0.462   0.018   0.047   0.935
E Y  1S78A                279    0.634 135.486  -1.204   0.018   0.047   0.935
E N  1S78A                280    0.453  66.363  -0.997   0.020   0.205   0.775
B Y  1S78A                281    0.138  29.405  -0.504   0.021   0.451   0.528
E L  1S78A                282    0.289  52.971  -0.590   0.023   0.655   0.322
B S  1S78A                283    0.238  27.929  -0.424   0.023   0.655   0.322
E T  1S78A                284    0.334  46.256  -0.199   0.021   0.279   0.699
E D  1S78A                285    0.677  97.613  -2.167   0.018   0.047   0.935
E V  1S78A                286    0.731 112.401  -0.134   0.005   0.015   0.979
E G  1S78A                287    0.423  33.314  -0.640   0.005   0.045   0.951
E S  1S78A                288    0.425  49.810   1.293   0.004   0.514   0.481
B C  1S78A                289    0.053   7.511  -0.858   0.002   0.816   0.182
E T  1S78A                290    0.281  39.002   0.665   0.011   0.918   0.071
E L  1S78A                291    0.401  73.496  -0.493   0.018   0.846   0.136
E V  1S78A                292    0.534  82.014   0.654   0.004   0.514   0.481
B C  1S78A                293    0.067   9.407  -0.248   0.004   0.085   0.910
E P  1S78A                294    0.564  80.060  -0.403   0.005   0.015   0.979
E L  1S78A                295    0.746 136.611  -0.815   0.003   0.003   0.994
E H  1S78A                296    0.569 103.537  -1.775   0.005   0.045   0.951
E N  1S78A                297    0.291  42.529  -0.225   0.005   0.336   0.660
B Q  1S78A                298    0.267  47.668  -0.907   0.004   0.616   0.381
E E  1S78A                299    0.407  71.190  -1.169   0.021   0.756   0.223
E V  1S78A                300    0.288  44.235  -1.340   0.021   0.756   0.223
E T  1S78A                301    0.432  59.932  -1.018   0.022   0.552   0.426
E A  1S78A                302    0.560  61.723  -1.304   0.022   0.359   0.619
E E  1S78A                303    0.665 116.193  -0.898   0.020   0.205   0.775
E D  1S78A                304    0.625  90.091  -1.683   0.018   0.047   0.935
E G  1S78A                305    0.475  37.351  -0.931   0.018   0.047   0.935
E T  1S78A                306    0.483  67.006  -0.817   0.021   0.279   0.699
E Q  1S78A                307    0.386  68.940  -0.579   0.004   0.514   0.481
E R  1S78A                308    0.464 106.210   0.114   0.021   0.756   0.223
B C  1S78A                309    0.159  22.380  -2.264   0.018   0.846   0.136
E E  1S78A                310    0.440  76.868  -0.449   0.023   0.655   0.322
E K  1S78A                311    0.533 109.741  -0.034   0.022   0.359   0.619
B C  1S78A                312    0.115  16.202  -2.425   0.020   0.205   0.775
E S  1S78A                313    0.566  66.323  -1.363   0.018   0.088   0.893
E K  1S78A                314    0.588 120.910  -1.993   0.018   0.019   0.964
E P  1S78A                315    0.535  75.874  -0.420   0.018   0.019   0.964
B C  1S78A                316    0.117  16.469  -1.900   0.018   0.047   0.935
E A  1S78A                317    0.478  52.643  -0.374   0.056   0.142   0.802
E R  1S78A                318    0.524 119.996  -0.970   0.020   0.205   0.775
E V  1S78A                319    0.316  48.569  -1.708   0.021   0.279   0.699
E C  1S78A                320    0.376  52.776  -1.330   0.021   0.451   0.528
E Y  1S78A                321    0.529 112.983  -1.126   0.021   0.451   0.528
E G  1S78A                322    0.397  31.260  -0.941   0.022   0.359   0.619
E L  1S78A                323    0.500  91.587  -1.429   0.021   0.279   0.699
E G  1S78A                324    0.515  40.562  -2.234   0.020   0.205   0.775
E M  1S78A                325    0.495  99.150  -1.384   0.021   0.279   0.699
E E  1S78A                326    0.518  90.477  -1.290   0.021   0.279   0.699
E H  1S78A                327    0.498  90.641  -1.023   0.020   0.205   0.775
E L  1S78A                328    0.495  90.635  -1.282   0.020   0.205   0.775
E R  1S78A                329    0.447 102.340  -0.899   0.021   0.279   0.699
E E  1S78A                330    0.371  64.779  -1.267   0.022   0.359   0.619
B V  1S78A                331    0.245  37.672  -1.034   0.021   0.451   0.528
E R  1S78A                332    0.446 102.157  -1.241   0.021   0.451   0.528
B A  1S78A                333    0.281  31.010  -2.050   0.022   0.359   0.619
E V  1S78A                334    0.378  58.114  -0.754   0.022   0.552   0.426
E T  1S78A                335    0.449  62.318  -0.944   0.022   0.552   0.426
E S  1S78A                336    0.408  47.782  -1.144   0.022   0.359   0.619
E A  1S78A                337    0.440  48.510  -1.518   0.021   0.279   0.699
E N  1S78A                338    0.583  85.293  -1.688   0.019   0.141   0.840
E I  1S78A                339    0.377  69.838  -1.236   0.018   0.047   0.935
E Q  1S78A                340    0.591 105.499  -0.993   0.018   0.047   0.935
E E  1S78A                341    0.350  61.110  -0.725   0.019   0.141   0.840
E F  1S78A                342    0.261  52.483  -0.954   0.019   0.141   0.840
E A  1S78A                343    0.525  57.888  -0.379   0.004   0.138   0.858
E G  1S78A                344    0.583  45.851   0.300   0.004   0.085   0.910
B C  1S78A                345    0.053   7.427  -1.001   0.004   0.138   0.858
E K  1S78A                346    0.450  92.668  -0.737   0.004   0.420   0.576
E K  1S78A                347    0.481  98.942  -0.455   0.023   0.655   0.322
E I  1S78A                348    0.263  48.710  -0.762   0.021   0.451   0.528
E F  1S78A                349    0.550 110.345  -1.991   0.004   0.138   0.858
E G  1S78A                350    0.505  39.743  -2.163   0.005   0.045   0.951
E S  1S78A                351    0.463  54.287  -2.580   0.004   0.197   0.799
B L  1S78A                352    0.214  39.257  -1.301   0.022   0.552   0.426
E A  1S78A                353    0.452  49.799  -0.988   0.021   0.756   0.223
B F  1S78A                354    0.125  25.047  -2.416   0.023   0.655   0.322
E L  1S78A                355    0.421  77.012  -1.567   0.021   0.451   0.528
B P  1S78A                356    0.278  39.406  -1.752   0.022   0.359   0.619
E E  1S78A                357    0.576 100.680  -1.212   0.022   0.359   0.619
E S  1S78A                358    0.368  43.094  -2.662   0.021   0.279   0.699
E F  1S78A                359    0.313  62.899  -1.497   0.022   0.359   0.619
E D  1S78A                360    0.625  90.077  -1.253   0.021   0.279   0.699
E G  1S78A                361    0.376  29.599  -1.395   0.020   0.205   0.775
E D  1S78A                362    0.506  72.886  -1.197   0.019   0.141   0.840
E P  1S78A                363    0.493  69.943  -1.603   0.019   0.141   0.840
E A  1S78A                364    0.466  51.375  -1.486   0.019   0.141   0.840
E S  1S78A                365    0.353  41.418  -1.283   0.020   0.205   0.775
E N  1S78A                366    0.493  72.204  -1.328   0.020   0.205   0.775
E T  1S78A                367    0.425  59.003  -1.323   0.020   0.205   0.775
E A  1S78A                368    0.400  44.047  -1.880   0.019   0.141   0.840
E P  1S78A                369    0.498  70.737  -1.627   0.019   0.141   0.840
E L  1S78A                370    0.375  68.626  -1.289   0.020   0.205   0.775
E Q  1S78A                371    0.433  77.352  -0.745   0.056   0.142   0.802
E P  1S78A                372    0.441  62.635  -1.508   0.052   0.084   0.864
E E  1S78A                373    0.594 103.824  -1.378   0.113   0.087   0.800
E Q  1S78A                374    0.395  70.636  -0.997   0.113   0.087   0.800
E L  1S78A                375    0.330  60.496  -0.796   0.184   0.043   0.773
E Q  1S78A                376    0.590 105.428  -1.007   0.113   0.087   0.800
E V  1S78A                377    0.303  46.633  -0.933   0.113   0.087   0.800
B F  1S78A                378    0.226  45.298  -1.200   0.056   0.142   0.802
E E  1S78A                379    0.427  74.614  -0.567   0.021   0.279   0.699
E T  1S78A                380    0.454  62.928  -0.102   0.022   0.359   0.619
B L  1S78A                381    0.098  18.035   0.022   0.022   0.552   0.426
E E  1S78A                382    0.484  84.502  -0.196   0.022   0.552   0.426
E E  1S78A                383    0.391  68.378  -0.737   0.021   0.451   0.528
B I  1S78A                384    0.194  35.909  -1.572   0.005   0.336   0.660
E T  1S78A                385    0.434  60.265  -1.837   0.005   0.262   0.733
E G  1S78A                386    0.429  33.747  -1.616   0.005   0.262   0.733
B Y  1S78A                387    0.252  53.831  -1.164   0.004   0.420   0.576
B L  1S78A                388    0.140  25.561  -0.500   0.023   0.655   0.322
E Y  1S78A                389    0.359  76.782  -0.511   0.018   0.846   0.136
B I  1S78A                390    0.182  33.633  -1.697   0.021   0.756   0.223
E S  1S78A                391    0.432  50.666  -1.573   0.021   0.451   0.528
E A  1S78A                392    0.573  63.101  -1.289   0.022   0.359   0.619
E W  1S78A                393    0.324  77.994  -0.662   0.022   0.359   0.619
E P  1S78A                394    0.482  68.382  -0.863   0.020   0.205   0.775
E D  1S78A                395    0.528  76.128  -1.241   0.019   0.141   0.840
E S  1S78A                396    0.460  53.889  -1.312   0.018   0.088   0.893
B L  1S78A                397    0.176  32.189  -1.464   0.018   0.088   0.893
E P  1S78A                398    0.538  76.385  -1.012   0.018   0.088   0.893
E D  1S78A                399    0.455  65.580  -2.119   0.018   0.047   0.935
E L  1S78A                400    0.311  56.962  -1.673   0.018   0.047   0.935
E S  1S78A                401    0.541  63.440  -1.307   0.018   0.088   0.893
B V  1S78A                402    0.229  35.228  -1.378   0.019   0.141   0.840
B F  1S78A                403    0.208  41.806  -1.683   0.018   0.088   0.893
E Q  1S78A                404    0.627 112.000  -0.864   0.018   0.088   0.893
E N  1S78A                405    0.579  84.839  -0.949   0.018   0.047   0.935
B L  1S78A                406    0.187  34.240  -0.788   0.018   0.088   0.893
E Q  1S78A                407    0.527  94.086  -0.703   0.005   0.336   0.660
E V  1S78A                408    0.409  62.817   0.086   0.004   0.616   0.381
B I  1S78A                409    0.198  36.611  -1.232   0.003   0.718   0.279
E R  1S78A                410    0.505 115.691  -0.798   0.022   0.552   0.426
E G  1S78A                411    0.387  30.425  -1.309   0.021   0.451   0.528
E R  1S78A                412    0.540 123.614  -0.288   0.022   0.552   0.426
E I  1S78A                413    0.415  76.738  -0.504   0.022   0.552   0.426
E L  1S78A                414    0.258  47.185  -0.930   0.022   0.359   0.619
E H  1S78A                415    0.510  92.842  -1.409   0.022   0.359   0.619
E N  1S78A                416    0.596  87.254  -2.311   0.019   0.141   0.840
E G  1S78A                417    0.529  41.640  -2.376   0.018   0.088   0.893
E A  1S78A                418    0.381  41.975  -0.986   0.022   0.359   0.619
B Y  1S78A                419    0.159  34.064  -1.332   0.022   0.552   0.426
B S  1S78A                420    0.122  14.345  -1.800   0.023   0.655   0.322
B L  1S78A                421    0.085  15.618  -1.089   0.018   0.846   0.136
B T  1S78A                422    0.160  22.261  -1.397   0.018   0.846   0.136
B L  1S78A                423    0.173  31.731  -1.069   0.023   0.655   0.322
E Q  1S78A                424    0.438  78.263  -1.096   0.021   0.451   0.528
B G  1S78A                425    0.235  18.518  -1.211   0.020   0.205   0.775
E L  1S78A                426    0.422  77.305  -1.151   0.018   0.088   0.893
E G  1S78A                427    0.546  42.986  -1.416   0.019   0.141   0.840
B I  1S78A                428    0.263  48.599  -0.902   0.020   0.205   0.775
E S  1S78A                429    0.346  40.539  -1.145   0.020   0.205   0.775
E W  1S78A                430    0.470 112.939  -1.832   0.020   0.205   0.775
E L  1S78A                431    0.427  78.202  -2.000   0.018   0.047   0.935
E G  1S78A                432    0.563  44.300  -2.000   0.018   0.047   0.935
E L  1S78A                433    0.381  69.816  -1.408   0.018   0.047   0.935
E R  1S78A                434    0.517 118.507  -1.140   0.018   0.088   0.893
E S  1S78A                435    0.506  59.303  -1.200   0.019   0.141   0.840
E L  1S78A                436    0.477  87.394  -1.459   0.021   0.279   0.699
E R  1S78A                437    0.305  69.822  -0.738   0.021   0.451   0.528
E E  1S78A                438    0.459  80.170  -1.044   0.021   0.451   0.528
B L  1S78A                439    0.114  20.910  -0.985   0.022   0.359   0.619
E G  1S78A                440    0.488  38.390  -1.632   0.021   0.279   0.699
E S  1S78A                441    0.635  74.410  -1.340   0.018   0.088   0.893
E G  1S78A                442    0.370  29.135  -2.385   0.053   0.043   0.903
E L  1S78A                443    0.456  83.475  -1.286   0.066   0.296   0.638
B A  1S78A                444    0.229  25.225  -1.104   0.021   0.756   0.223
E L  1S78A                445    0.290  53.154  -0.891   0.018   0.846   0.136
B I  1S78A                446    0.286  52.854  -1.600   0.021   0.756   0.223
E H  1S78A                447    0.421  76.507  -1.535   0.022   0.552   0.426
E H  1S78A                448    0.465  84.583  -1.948   0.020   0.205   0.775
E N  1S78A                449    0.345  50.464  -2.026   0.019   0.141   0.840
E T  1S78A                450    0.453  62.887  -1.503   0.020   0.205   0.775
E H  1S78A                451    0.441  80.236  -1.597   0.021   0.279   0.699
B L  1S78A                452    0.238  43.505  -1.102   0.021   0.279   0.699
B C  1S78A                453    0.120  16.904  -2.783   0.021   0.279   0.699
E F  1S78A                454    0.340  68.238  -1.369   0.022   0.359   0.619
E V  1S78A                455    0.448  68.796  -1.497   0.022   0.359   0.619
E H  1S78A                456    0.439  79.909  -1.406   0.022   0.359   0.619
E T  1S78A                457    0.466  64.620  -1.747   0.020   0.205   0.775
E V  1S78A                458    0.514  78.940  -1.803   0.018   0.088   0.893
E P  1S78A                459    0.594  84.345  -0.808   0.018   0.047   0.935
E W  1S78A                460    0.268  64.454  -0.707   0.018   0.047   0.935
E D  1S78A                461    0.503  72.526  -1.206   0.019   0.141   0.840
E Q  1S78A                462    0.389  69.493  -0.920   0.021   0.279   0.699
B L  1S78A                463    0.225  41.216  -0.896   0.022   0.359   0.619
E F  1S78A                464    0.392  78.614  -1.582   0.021   0.451   0.528
E R  1S78A                465    0.494 113.057  -1.369   0.022   0.359   0.619
E N  1S78A                466    0.373  54.666  -1.341   0.019   0.141   0.840
E P  1S78A                467    0.636  90.192  -1.176   0.018   0.088   0.893
E H  1S78A                468    0.495  90.059  -0.584   0.018   0.088   0.893
E Q  1S78A                469    0.365  65.260  -0.988   0.004   0.138   0.858
E A  1S78A                470    0.320  35.297  -1.276   0.022   0.359   0.619
E L  1S78A                471    0.364  66.722  -0.857   0.023   0.655   0.322
E L  1S78A                472    0.352  64.506  -1.308   0.023   0.655   0.322
B H  1S78A                473    0.221  40.127  -1.407   0.021   0.451   0.528
E T  1S78A                474    0.376  52.137  -1.190   0.021   0.279   0.699
E A  1S78A                475    0.559  61.591  -2.091   0.019   0.141   0.840
E N  1S78A                476    0.572  83.814  -2.096   0.018   0.047   0.935
E R  1S78A                477    0.449 102.844  -1.084   0.019   0.141   0.840
E P  1S78A                478    0.390  55.298  -0.942   0.021   0.279   0.699
E E  1S78A                479    0.411  71.819  -0.821   0.069   0.386   0.545
E D  1S78A                480    0.490  70.667  -0.939   0.069   0.386   0.545
E E  1S78A                481    0.446  77.846  -0.422   0.066   0.296   0.638
E C  1S78A                482    0.386  54.124  -0.572   0.056   0.142   0.802
E V  1S78A                483    0.485  74.575  -0.160   0.052   0.084   0.864
E G  1S78A                484    0.559  44.001  -0.246   0.018   0.088   0.893
E E  1S78A                485    0.566  98.950   0.593   0.019   0.141   0.840
E G  1S78A                486    0.329  25.869  -0.424   0.005   0.262   0.733
E L  1S78A                487    0.437  80.088  -0.145   0.004   0.197   0.799
E A  1S78A                488    0.510  56.180   0.183   0.004   0.197   0.799
B C  1S78A                489    0.067   9.351  -2.022   0.004   0.085   0.910
E H  1S78A                490    0.434  78.963  -0.074   0.018   0.019   0.964
E Q  1S78A                491    0.724 129.306  -1.194   0.018   0.019   0.964
E L  1S78A                492    0.502  91.843   0.051   0.018   0.019   0.964
B C  1S78A                493    0.068   9.575  -2.453   0.018   0.047   0.935
E A  1S78A                494    0.578  63.740  -0.412   0.019   0.141   0.840
E R  1S78A                495    0.619 141.659  -0.885   0.004   0.138   0.858
E G  1S78A                496    0.428  33.668  -0.977   0.004   0.138   0.858
E H  1S78A                497    0.488  88.840  -1.222   0.004   0.197   0.799
B C  1S78A                498    0.162  22.717  -2.511   0.005   0.336   0.660
E W  1S78A                499    0.470 113.083  -0.978   0.005   0.262   0.733
E G  1S78A                500    0.555  43.694  -1.517   0.005   0.045   0.951
E P  1S78A                501    0.619  87.794  -1.542   0.005   0.015   0.979
E G  1S78A                502    0.489  38.469  -1.142   0.018   0.019   0.964
E P  1S78A                503    0.459  65.104  -0.289   0.018   0.047   0.935
E T  1S78A                504    0.535  74.149  -0.602   0.018   0.088   0.893
E Q  1S78A                505    0.333  59.474   0.378   0.019   0.141   0.840
B C  1S78A                506    0.073  10.235  -3.041   0.022   0.359   0.619
B V  1S78A                507    0.249  38.225  -0.571   0.021   0.451   0.528
E N  1S78A                508    0.557  81.530  -0.245   0.021   0.451   0.528
B C  1S78A                509    0.129  18.055  -0.972   0.021   0.279   0.699
E S  1S78A                510    0.529  61.964  -0.867   0.019   0.141   0.840
E Q  1S78A                511    0.529  94.462  -1.012   0.019   0.141   0.840
B F  1S78A                512    0.240  48.088  -2.152   0.020   0.205   0.775
E L  1S78A                513    0.325  59.526  -1.039   0.021   0.451   0.528
E R  1S78A                514    0.441 100.897  -1.068   0.021   0.451   0.528
E G  1S78A                515    0.495  38.941  -1.436   0.019   0.141   0.840
E Q  1S78A                516    0.510  91.015  -1.284   0.004   0.085   0.910
E E  1S78A                517    0.462  80.729  -0.157   0.021   0.279   0.699
B C  1S78A                518    0.204  28.670  -0.455   0.021   0.451   0.528
E V  1S78A                519    0.447  68.735  -0.282   0.022   0.359   0.619
E E  1S78A                520    0.543  94.897  -0.524   0.022   0.359   0.619
E E  1S78A                521    0.522  91.141   0.960   0.022   0.359   0.619
B C  1S78A                522    0.027   3.861  -1.153   0.019   0.141   0.840
E R  1S78A                523    0.522 119.538  -0.369   0.019   0.141   0.840
E V  1S78A                524    0.398  61.219  -1.416   0.004   0.085   0.910
E L  1S78A                525    0.476  87.229  -1.639   0.018   0.088   0.893
E Q  1S78A                526    0.536  95.658  -1.726   0.018   0.088   0.893
E G  1S78A                527    0.432  33.967  -1.508   0.019   0.141   0.840
E L  1S78A                528    0.454  83.036  -1.079   0.019   0.141   0.840
E P  1S78A                529    0.311  44.188  -0.975   0.020   0.205   0.775
E R  1S78A                530    0.535 122.446  -0.920   0.020   0.205   0.775
E E  1S78A                531    0.517  90.320  -1.354   0.021   0.279   0.699
E Y  1S78A                532    0.222  47.335  -1.001   0.020   0.205   0.775
E V  1S78A                533    0.486  74.698  -1.126   0.052   0.084   0.864
E N  1S78A                534    0.636  93.169  -1.214   0.018   0.047   0.935
E A  1S78A                535    0.635  69.988  -0.877   0.018   0.019   0.964
E R  1S78A                536    0.475 108.775   0.313   0.018   0.019   0.964
E H  1S78A                537    0.446  81.146   1.397   0.019   0.141   0.840
B C  1S78A                538    0.087  12.229  -1.387   0.021   0.279   0.699
E L  1S78A                539    0.427  78.165   0.427   0.021   0.279   0.699
E P  1S78A                540    0.577  81.933   0.599   0.019   0.141   0.840
B C  1S78A                541    0.101  14.152  -0.495   0.053   0.043   0.903
E H  1S78A                542    0.432  78.508   0.458   0.115   0.016   0.868
E P  1S78A                543    0.693  98.379   0.254   0.257   0.016   0.727
E E  1S78A                544    0.527  92.119   0.782   0.268   0.043   0.689
B C  1S78A                545    0.043   6.023  -2.168   0.278   0.093   0.628
E Q  1S78A                546    0.480  85.799   0.299   0.199   0.152   0.649
E P  1S78A                547    0.586  83.125  -0.276   0.118   0.150   0.732
E Q  1S78A                548    0.315  56.223  -0.937   0.056   0.142   0.802
E N  1S78A                549    0.566  82.921  -2.128   0.020   0.205   0.775
E G  1S78A                550    0.502  39.539  -0.703   0.020   0.205   0.775
E S  1S78A                551    0.519  60.803  -0.689   0.004   0.197   0.799
E V  1S78A                552    0.442  67.935  -0.591   0.005   0.262   0.733
E T  1S78A                553    0.376  52.193  -0.202   0.005   0.262   0.733
B C  1S78A                554    0.065   9.112  -2.353   0.005   0.336   0.660
E F  1S78A                555    0.543 108.920  -0.870   0.005   0.336   0.660
E G  1S78A                556    0.391  30.811  -1.459   0.004   0.085   0.910
E P  1S78A                557    0.545  77.335  -1.290   0.005   0.015   0.979
E E  1S78A                558    0.547  95.491  -1.305   0.003   0.003   0.994
E A  1S78A                559    0.499  55.001  -0.495   0.018   0.019   0.964
E D  1S78A                560    0.581  83.751  -0.909   0.058   0.017   0.925
E Q  1S78A                561    0.377  67.279   0.812   0.056   0.142   0.802
B C  1S78A                562    0.063   8.859  -1.674   0.066   0.296   0.638
E V  1S78A                563    0.279  42.913   0.291   0.074   0.484   0.442
E A  1S78A                564    0.516  56.874  -0.146   0.069   0.386   0.545
B C  1S78A                565    0.169  23.728  -0.609   0.064   0.216   0.721
E A  1S78A                566    0.454  50.020  -0.400   0.056   0.142   0.802
E H  1S78A                567    0.457  83.201  -1.073   0.019   0.141   0.840
B Y  1S78A                568    0.241  51.544  -1.114   0.019   0.141   0.840
E K  1S78A                569    0.372  76.603  -0.868   0.019   0.141   0.840
E D  1S78A                570    0.497  71.546  -1.268   0.018   0.088   0.893
E P  1S78A                571    0.634  90.007  -0.971   0.005   0.015   0.979
E P  1S78A                572    0.498  70.709  -1.549   0.005   0.015   0.979
E F  1S78A                573    0.394  78.975   0.962   0.004   0.420   0.576
B C  1S78A                574    0.034   4.746  -1.282   0.002   0.816   0.182
E V  1S78A                575    0.280  42.990   0.713   0.018   0.846   0.136
E A  1S78A                576    0.386  42.515  -0.760   0.018   0.846   0.136
E R  1S78A                577    0.476 108.912   1.185   0.021   0.451   0.528
B C  1S78A                578    0.039   5.490  -0.436   0.005   0.045   0.951
E P  1S78A                579    0.478  67.800  -0.414   0.005   0.015   0.979
E S  1S78A                580    0.729  85.392  -0.523   0.005   0.015   0.979
E G  1S78A                581    0.462  36.344  -2.124   0.018   0.047   0.935
E V  1S78A                582    0.333  51.167  -0.532   0.021   0.279   0.699
B K  1S78A                583    0.252  51.775  -1.070   0.022   0.552   0.426
E P  1S78A                584    0.454  64.437  -1.037   0.021   0.451   0.528
E D  1S78A                585    0.441  63.519  -1.728   0.021   0.279   0.699
E L  1S78A                586    0.577 105.612  -1.937   0.019   0.141   0.840
E S  1S78A                587    0.620  72.652  -2.111   0.020   0.205   0.775
E Y  1S78A                588    0.453  96.763  -0.632   0.021   0.279   0.699
E M  1S78A                589    0.344  68.854  -0.688   0.022   0.359   0.619
E P  1S78A                590    0.363  51.524  -0.772   0.022   0.552   0.426
E I  1S78A                591    0.351  64.842  -1.498   0.023   0.655   0.322
E W  1S78A                592    0.411  98.725  -1.145   0.022   0.552   0.426
E K  1S78A                593    0.399  82.157  -0.806   0.021   0.451   0.528
E F  1S78A                594    0.288  57.882  -0.909   0.021   0.279   0.699
E P  1S78A                595    0.469  66.579  -0.978   0.019   0.141   0.840
E D  1S78A                596    0.517  74.500  -1.051   0.018   0.019   0.964
E E  1S78A                597    0.644 112.419  -0.352   0.018   0.019   0.964
E E  1S78A                598    0.675 117.905   0.141   0.003   0.003   0.994
E G  1S78A                599    0.445  35.021   0.647   0.005   0.015   0.979
E A  1S78A                600    0.450  49.579   1.925   0.020   0.205   0.775
B C  1S78A                601    0.074  10.362  -1.187   0.004   0.420   0.576
E Q  1S78A                602    0.473  84.460   1.217   0.021   0.451   0.528
E P  1S78A                603    0.600  85.197   1.411   0.021   0.279   0.699
B C  1S78A                604    0.130  18.210   0.044   0.018   0.047   0.935
E P  1S78A                605    0.491  69.701   0.505   0.058   0.017   0.925
E I  1S78A                606    0.654 121.027  -0.190   0.058   0.017   0.925
E N  1S78A                607    0.534  78.222   0.797   0.053   0.043   0.903
B C  1S78A                608    0.070   9.800  -0.249   0.052   0.084   0.864
E T  1S78A                609    0.479  66.437   0.505   0.064   0.216   0.721
E H  1S78A                610    0.524  95.352   0.655   0.066   0.296   0.638
E S  1S78A                611    0.420  49.283   0.274   0.069   0.386   0.545
B C  1S78A                612    0.045   6.360  -1.341   0.022   0.359   0.619
E V  1S78A                613    0.637  97.938  -0.207   0.005   0.262   0.733
E D  1S78A                614    0.346  49.859  -1.481   0.005   0.045   0.951
E L  1S78A                615    0.579 106.070  -1.406   0.005   0.015   0.979
E D  1S78A                616    0.535  77.094   0.015   0.003   0.003   0.994
E D  1S78A                617    0.592  85.322  -0.484   0.003   0.003   0.994
E K  1S78A                618    0.673 138.333   0.356   0.003   0.003   0.994
E G  1S78A                619    0.565  44.497   1.488   0.005   0.015   0.979
B C  1S78A                620    0.067   9.435  -0.793   0.005   0.045   0.951
E P  1S78A                621    0.498  70.666   1.090   0.004   0.197   0.799
E A  1S78A                622    0.620  68.280   0.955   0.020   0.205   0.775
E E  1S78A                623    0.333  58.245  -0.091   0.019   0.141   0.840
E Q  1S78A                624    0.717 128.110   0.429   0.003   0.003   0.994

Explain the output.  Go back.
```

---
